# Optic Disc Characteristics in Children Born Preterm With and Without ROP: Results From the Gutenberg Prematurity Eye Study Young (GPESY)

**DOI:** 10.1167/iovs.66.13.21

**Published:** 2025-10-09

**Authors:** Achim Fieß, Sandra Gißler, Stephanie Grabitz, Eva Mildenberger, Timo Uphaus, Marianne Hahn, Norbert Pfeiffer, Alica Hartmann, Alexander K. Schuster

**Affiliations:** 1 Department of Ophthalmology, University Medical Center of the Johannes Gutenberg University Mainz, Mainz, Germany; 2 Division of Neonatology, Department of Pediatrics, University Medical Center of the Johannes Gutenberg University Mainz, Mainz, Germany; 3 Department of Neurology, Focus Program Translational Neuroscience (FTN) and Immunotherapy (FZI), Rhine Main Neuroscience Network (rmn2), University Medical Center of the Johannes Gutenberg University Mainz, Mainz, Germany; 4 Department of Neurology and Focus Program Translational Neuroscience (FTN), Rhine Main Neuroscience Network (rmn2), University Medical Center of the Johannes Gutenberg University Mainz, Mainz, Germany

**Keywords:** epidemiology, preterm birth, VCDR, MRW, RNFL

## Abstract

**Purpose:**

We investigated optic nerve head morphology in children born preterm with and without retinopathy of prematurity (ROP), focusing on peripapillary retinal nerve fiber layer (pRNFL) thickness, minimal rim width (MRW), Bruch's membrane opening (BMO), and vertical cup-to-disc ratio (vCDR) in relation to perinatal factors (gestational age [GA], birth weight [BW], perinatal adverse events [PAE]).

**Methods:**

This prospective observational cohort included 793 former preterm children aged four to 17 years, stratified into late preterm (GA 33–36 weeks), moderate preterm (GA 29–32 weeks), extreme preterm (GA ≤28 weeks), preterm with untreated ROP, preterm with ROP treatment, and full-term controls (GA ≥37 weeks). Effects of perinatal factors on pRNFL, MRW, BMO area, and vCDR were evaluated.

**Results:**

Extremely preterm children had a thinner pRNFL (β = −9.25, *P* < 0.001), except temporally. ROP-treated children showed thicker temporal (β = 43.31, *P* < 0.001) and inferotemporal (β = 20.97, *P* = 0.04) pRNFL but thinner superonasal sectors. PAE (β = −7.68, *P* < 0.001) and maternal smoking (temporal β = −12.02, *P* = 0.003) were associated with thinner pRNFL, whereas breastfeeding was linked to thicker pRNFL (β = 2.25, *P* = 0.003). MRW was thinner in extremely preterm infants, particularly inferiorly (inferotemporal β = −30.36, inferonasal β = −28.85, all *P* ≤ 0.02). VCDR was larger in extreme (β = 0.05, *P* = 0.001) and moderate (β = 0.04, *P* = 0.004) preterms. BMO area showed no associations.

**Conclusions:**

Prematurity was associated with thinner pRNFL, smaller MRW, and larger vCDR. ROP treatment was linked to thicker temporal pRNFL and thicker MRW. PAE and maternal smoking were associated with thinner pRNFL, whereas breastfeeding correlated with greater thickness.

Preterm birth, defined as delivery before 37 weeks of gestation,^1^ affects neurodevelopment and is associated with changes in optic nerve head morphology.^2^ Several studies have assessed the optic nerve head characteristics in children and adults born preterm and mainly analyzed the thickness of the (peripapillary) retinal nerve fiber layer (pRNFL),^3^^–^^5^ cup-to-disc ratio (CDR),^6^^–^^9^ and optic disc size.^10^^,^^11^ The assessment of other optic disc characteristics such as minimal rim width (MRW)^12^ and opening of the Bruch's-membrane (BMO) remain scarce, although these provide additional information on the status of the optic nerve head. This is important because changes in the anatomy of the optic nerve head, especially found in preterm children, may raise suspicion of glaucoma, which may lead to lifelong antiglaucomatous medication.^13^ To date, one study by Lee et al.^14^ has assessed BMO and MRW in 120 school-aged children born preterm and full-term, including 42 children born full-term, 41 children born preterm without retinopathy of prematurity (ROP), 16 children with ROP treated with intravitreal bevacizumab, and 21 children with ROP treated with laser. They found that laser-treated children had a greater MRW and thicker temporal pRNFL than children born full-term or preterm without ROP treatment.^14^ Wenner et al.^13^ focused on pRNFL, CDR, and rim width measures in 55 children born preterm (gestational age [GA] ≤ 36 weeks, mean age 10 ± 2.5 years) with and without brain injury. Both studies involved small sample sizes and participants with ROP, but treatment assessment was only included by Lee et al.^14^

The aim of the Gutenberg Prematurity Eye Study Young (GPESY) was to assess the optic nerve head morphology of a large cohort of children aged four to 17 years with and without ROP including a comprehensive analysis of pRNFL-thickness, MRW, size of BMO area, and several morphological features of the optic disc such as the vertical CDR (vCDR) in relation to perinatal information such as GA, birth weight percentile, perinatal adverse events, maternal smoking during pregnancy and breastfeeding.

## Material and Methods

### Study Population

The GPESY was conducted at the University Medical Center of the Johannes Gutenberg University Mainz (UMCM) in Germany and involved children and adolescents aged four to 17 years born preterm or at term (between 2003 and 2018). It is a retrospective cohort study with a prospective ophthalmologic examination and an extension to the Gutenberg Prematurity Study (GPS) in adults.^4^^,^^6^^,^^15^^–^^22^ The perinatal history was assessed retrospectively by reviewing the medical record data stored in the UMCM, whereas the prospective part consisted of an extensive ophthalmologic examination. Potential participants were selected by an algorithm inviting every individual born at a GA at birth ≤32 weeks and every third individual born at a GA of 33 to 36 weeks. Additionally, eight individuals born full-term (four male individuals and four female individuals from the beginning of each month of the calendar) with a birthweight between the tenth and ninetieth percentile were recruited as controls (see Supplementary Figure S1). Written informed consent was obtained from all participants and their legal guardians before their study entry. The participants were examined, and their medical history was recorded between 2022 and 2023. The GPESY complies with Good Clinical Practice (GCP), Good Epidemiological Practice (GEP), and the ethical principles of the Declaration of Helsinki. The study protocol and documents were approved by the local ethics committee of the Medical Chamber of Rhineland-Palatinate, Germany (reference no. 2021-15830; original vote: May 5, 2021, latest update: January 19, 2022).

### Assessment of Prenatal, Perinatal, and Postnatal Medical History

Medical records at the UMCM were reviewed for the following data: GA (weeks), birth weight (kg), presence of ROP, stage of ROP, ROP treatment, placental insufficiency, preeclampsia, breastfeeding, maternal smoking during pregnancy, and perinatal adverse events. Birth weight percentiles were calculated according to Voigt et al,^23^ based on data from a newborn cohort in the Federal Republic of Germany.

### Categorization

Participants were categorized into six groups, consisting of term-born children with a GA at birth ≥37 completed weeks (group 1), participants born preterm with a GA of 33 to 36 weeks without ROP (group 2), participants born preterm with a GA of 29 to 32 weeks without ROP (group 3), participants born preterm with a GA ≤28 weeks without ROP (group 4), participants born preterm with a GA ≤32 and nontreated ROP (group 5), and those with treated ROP (group 6). In the case in which only one eye was affected with ROP, the other non-ROP eye was excluded from the analysis.

### Ophthalmologic Examination

The detailed ophthalmologic examination included testing of distant corrected visual acuity (DCVA) with an ARK 1s (NIDEK; Oculus, Wetzlar, Germany) and the measurement of axial length with a LenStar (Lenstar LS900; Haag-Streit, Bern, Switzerland). Additionally, the intraocular pressure was measured with a non-contact tonometer (NT 2000; Nidek Co., Tokyo, Japan). The visual acuity was converted from decimal to logMAR.^24^

### Optical Coherence Tomography

Spectral-domain optical coherence tomography (SD-OCT) of the peripapillary region was conducted using a peripapillary scan of 12° diameter centered on the optic disc with eye-tracking and standard 7.7 mm corneal curvature and ametropia of 0 diopters (D). We performed automated layer segmentation using the Heidelberg Eye Explorer Software tool (HEYEX, version 6.13.3.0). Global and sectorial pRNFL thickness (global, superonasal, nasal, inferonasal, inferotemporal, temporal, superotemporal) were automatically calculated by the software algorithm. Ocular magnification adjustments were made by taking into account the corneal curvature and spherical equivalent.^25^^,^^26^ Furthermore, 24 high-resolution optic nerve head radial scans and three circle scans were obtained using the Glaucoma Module Premier Edition software (version 6.10; Heidelberg Engineering Inc., Heidelberg, Germany). The BMO-MRW was defined as the shortest distance from the BMO to the internal limiting membrane and was automatically calculated with the HEYEX software (see Supplementary Fig. S3).^27^ The image quality of OCT scans was evaluated regarding centration and focus as well as accurate segmentation. Scans that did not meet these criteria were classified as invalid. Peripapillary RNFL layers were segmented manually in the case of missegmentation by the software, with the images excluded if segmentations could not be corrected or the quality was poor (<15 db).

### Fundus Photography and Optic Disc Measurements

Fundus photography (Visucam PRO NM; Zeiss, Oberkochen, Germany) was performed in a darkened room with mydriatic pupils, including 30° and 45° images of the optic nerve head. The right eye was imaged first, and regular data quality control was conducted. The discrepancy between the number of participants reported in the characteristics section and those included in the tables of fundus image measurements reflects the number of images that were unsuitable for further evaluation (Tables 1, 2).

**Table 1.** Characteristics of the Study Sample (*n* = 793) Stratified by Group

	Group 1 GA **≥** 37 Wks	Group 2 GA 33–36 Wks No ROP	Group 3 GA 29–32 Wks No ROP	Group 4 GA **≤** 28 Wks No ROP	Group 5 GA **≤** 32 Wks ROP Without Treatment	Group 6 GA **≤** 32 Wks ROP With Treatment
Participants (n)/eyes (n)	257/502	240/474	164/318	70/136	53/99	9/17
Sex (female)	137 (53.3)	118 (49.2)	86 (52.4)	40 (57.1)	32 (60.4)	5 (55.6)
Age (y), mean (SD)	11.25 (3.60)	11.20 (3.75)	11.72 (3.89)	11.07 (3.79)	12.62 (5.11)	11.78 (3.73)
Gestational age (weeks), mean (SD)	38.98 (1.28)	34.64 (1.03)	30.88 (1.06)	26.21 (1.44)	26.68 (2.38)	24.33 (0.87)
Birth weight (kg), mean (SD)	3.37 (0.40)	2.34 (0.41)	1.57 (0.35)	0.84 (0.24)	0.96 (0.36)	0.66 (0.23)
Birth weight <1500*g*	0 (0.0%)	7 (2.9%)	72 (43.9%)	70 (100.0%)	50 (94.3%)	9 (100.0%)
Birth weight <1000*g*	0 (0.0%)	0 (0.0%)	9 (5.5%)	52 (74.3%)	33 (62.3%)	8 (88.9%)
Birth weight percentile, mean (SD)	44.71 (24.74)	34.02 (22.83)	40.93 (22.28)	36.47 (25.94)	43.58 (27.97)	20.00 (27.44)
ROP	0 (0.0%)	0 (0.0%)	0 (0.0%)	0 (0.0%)	53 (100.0%)	9 (100.0%)
ROP Stage						
1	0	0	0	0	27	0
2	0	0	0	0	14	1
3	0	0	0	0	12	7
4	0	0	0	0	0	1
ROP plus disease	0 (0.0%)	0 (0.0%)	0 (0.0%)	0 (0.0%)	3 (5.7%)	4 (44.4%)
Perinatal adverse events^*^	0 (0.0%)	1 (0.4%)	7 (4.3%)	26 (37.1%)	22 (41.5%)	9 (100.0%)
IVH	0 (0.0%)	0 (0.0%)	0 (0.0%)	7 (10.0%)	3 (5.7%)	1 (11.1%)
NEC	0 (0.0%)	1 (0.4%)	4 (2.4%)	3 (4.3%)	2 (3.8%)	2 (22.2%)
BPD	0 (0.0%)	0 (0.0%)	3 (1.8%)	23 (32.9%)	21 (39.6%)	9 (100.0%)
Pre-eclampsia	4 (1.6%)	21 (8.8%)	29 (17.7%)	11 (15.7%)	11 (20.8%)	1 (11.1%)
Placental insufficiency	0 (0.0%)	8 (3.3%)	6 (3.7%)	5 (7.1%)	3 (5.7%)	1 (11.1%)
HELLP Syndrome (yes)	0 (0.0%)	8 (3.3%)	18 (11.0%)	6 (8.6%)	2 (3.8%)	0 (0.0%)
Gestational diabetes	32 (12.5%)	19 (7.9%)	19 (11.6%)	3 (4.3%)	3 (5.7%)	0 (0.0%)
Maternal smoking during pregnancy	12 (4.7%)	7 (2.9%)	7 (4.3%)	5 (7.1%)	7 (13.2%)	2 (22.2%)
Breastfeeding	222 (86.4%)	181 (75.4%)	115 (70.1%)	47 (67.1%)	24 (45.3%)	1 (11.1%)
Ocular parameters						
Visual acuity (LogMAR)						
OD, median [IQR]	0.00 [0.00, 0.10]	0.00 [0.00, 0.10]	0.00 [0.00, 0.10]	0.00 [0.00, 0.10]	0.00 [0.00, 0.20]	0.20 [0.20, 0.20]
OS, median [IQR]	0.00 [0.00, 0.10]	0.10 [0.00, 0.20]	0.05 [0.00, 0.10]	0.00 [0.00, 0.10]	0.00 [0.00, 0.20]	0.40 [0.10, 0.44]
Spherical equivalent (D)						
OD, mean (SD)	0.26 (1.42)	0.31 (1.47)	0.40 (1.62)	0.24 (2.05)	0.41 (2.10)	−1.54 (2.95)
OS, mean (SD)	0.24 (1.55)	0.34 (1.55)	0.47 (1.80)	0.34 (2.04)	0.77 (1.56)	−1.33 (3.24)
Intraocular pressure (mm Hg)						
OD, mean (SD)	17.00 (3.08)	16.36 (2.71)	16.28 (3.11)	18.02 (3.89)	17.25 (4.29)	17.34 (3.68)
OS, mean (SD)	16.38 (2.91)	15.80 (2.79)	15.70 (2.99)	17.13 (3.35)	16.67 (4.88)	16.89 (4.38)

BPD, bronchopulmonary dysplasia (any grade); D, diopters; g, grams; GA, gestational age; HELLP, hemolysis, elevated liver enzymes, low platelet; IQR, interquartile range; IVH, intraventricular hemorrhage (any grade); n, number; NEC, necrotizing enterocolitis (any grade); OD, right eye; OS, left eye; ROP, retinopathy of prematurity; SD, standard deviation; wks, weeks; y, years.

* Perinatal adverse events were defined as the occurrence of intraventricular hemorrhage (at least grade 3 or parenchymal hemorrhage) or occurrence of necrotizing enterocolitis and/or bronchopulmonary dysplasia (+moderate or severe).

**Table 2.** Summary of the Retinal Nerve Fiber Layer, Bruch's Membrane Opening, and Minimum Rim Width Parameters of Each Study Group (Eyes = 1313)

	Group 1 GA **≥** 37 Wks	Group 2 GA 33–36 Wks No ROP	Group 3 GA 29–32 Wks No ROP	Group 4 GA **≤** 28 Wks No ROP	Group 5 GA **≤** 32 Wks ROP Without Treatment	Group 6 GA **≤** 32 Wks ROP With Treatment	*P* Value
Eyes (n)							
OD	224	205	142	57	36	5	
OS	218	195	133	55	38	5	
pRNFL (µm) (12° circle-scan)							
Global, mean (SD)	102.55 (9.93)	101.77 (9.90)	100.42 (10.20)	93.12 (12.92)	94.43 (9.30)	93.51 (13.36)	<0.001
Superotemporal, mean (SD)	148.26 (20.38)	149.19 (21.94)	148.24 (22.62)	136.92 (22.42)	138.07 (21.87)	123.33 (19.39)	<0.001
Temporal, mean (SD)	73.42 (13.06)	72.19 (12.01)	70.15 (12.36)	66.35 (12.83)	66.08 (10.97)	102.94 (33.88)	<0.001
Inferotemporal, mean (SD)	145.81 (18.05)	144.52 (19.52)	145.76 (21.14)	133.33 (26.75)	133.80 (19.31)	151.02 (27.85)	<0.001
Inferonasal, mean (SD)	114.73 (25.52)	111.69 (25.16)	110.31 (25.88)	102.73 (26.58)	102.27 (26.15)	80.47 (30.52)	<0.001
Nasal, mean (SD)	72.44 (16.10)	71.16 (15.53)	68.69 (15.67)	64.58 (15.35)	66.69 (18.52)	54.38 (14.09)	<0.001
Superonasal, mean (SD)	120.08 (22.72)	122.05 (24.32)	121.49 (22.20)	110.33 (24.25)	115.48 (25.71)	78.96 (13.99)	<0.001
BMO area (mm^2^), mean (SD)	2.01 (0.43)	2.03 (0.36)	2.12 (0.48)	2.12 (0.38)	1.94 (0.42)	1.68 (0.29)	0.004
MRW (µm)							
Global, (SD)	380.39 (57.55)	371.88 (49.99)	384.63 (65.07)	345.32 (66.38)	362.30 (71.71)	424.28 (61.95)	<0.001
Superotemporal, mean (SD)	361.54 (68.85)	348.71 (65.26)	367.64 (71.81)	325.92 (56.18)	338.71 (69.20)	423.37 (50.01)	<0.001
Temporal, mean (SD)	272.28 (53.86)	265.26 (47.79)	273.63 (46.20)	250.52 (53.88)	272.01 (72.46)	362.27 (64.90)	<0.001
Inferotemporal, mean (SD)	416.65 (66.35)	404.51 (56.44)	420.52 (62.96)	369.12 (67.04)	394.74 (62.74)	469.70 (53.29)	<0.001
Inferonasal, mean (SD)	469.02 (66.13)	460.37 (64.89)	472.35 (77.89)	421.79 (89.23)	440.10 (84.97)	491.82 (50.13)	<0.001
Nasal, mean (SD)	411.37 (67.42)	406.22 (60.67)	414.61 (88.56)	376.16 (87.02)	387.33 (89.09)	421.10 (83.26)	0.001
Superonasal, mean (SD)	432.36 (78.50)	419.37 (72.41)	445.34 (90.89)	392.92 (78.65)	410.00 (85.01)	460.71 (67.89)	<0.001
Optic disc characteristics							
Eyes OD (n)	240	226	159	69	48	9	
Vertical disc length (mm)	1.73 (0.18)	1.74 (0.17)	1.75 (0.18)	1.76 (0.20)	1.71 (0.19)	1.78 (0.19)	0.680
Vertical cup length (mm)	0.48 (0.26)	0.50 (0.28)	0.53 (0.29)	0.62 (0.32)	0.56 (0.30)	0.47 (0.27)	0.008
VCDR [IQR]	0.26 [0.15, 0.38]	0.26 [0.16, 0.38]	0.28 [0.19, 0.42]	0.34 [0.24, 0.47]	0.34 [0.20, 0.42]	0.27 [0.21, 0.39]	0.013
Longer disc diameter (mm)	1.73 (0.18)	1.73 (0.17)	1.74 (0.18)	1.75 (0.20)	1.70 (0.19)	1.80 (0.28)	0.608
Shorter disc diameter (mm)	1.54 (0.16)	1.56 (0.16)	1.55 (0.16)	1.57 (0.19)	1.52 (0.19)	1.55 (0.20)	0.619
Optic disc area (mm^2^)	2.12 (0.43)	2.14 (0.41)	2.14 (0.42)	2.18 (0.50)	2.04 (0.47)	2.23 (0.63)	0.668
Torsion angle (absolute)°, [IQR]	17.00 [10.25, 27.50]	17.00 [9.50, 28.00]	15.00 [7.00, 22.00]	12.50 [9.00, 18.50]	14.00 [8.00, 24.50]	11.00 [2.00, 14.00]	0.012
Tilted	10 (4.8%)	11 (5.2%)	8 (5.7%)	3 (4.8%)	2 (4.8%)	0 (0.0%)	0.989
Torted	121 (57.6%)	116 (54.0%)	70 (48.3%)	24 (36.4%)	18 (41.9%)	2 (22.2%)	0.011
Eyes OS	221	211	140	57	46	7	
Vertical disc length (mm)	1.73 (0.17)	1.73 (0.18)	1.74 (0.18)	1.72 (0.15)	1.71 (0.18)	1.87 (0.37)	0.340
Vertical cup length (mm)	0.47 (0.26)	0.49 (0.28)	0.53 (0.27)	0.58 (0.29)	0.57 (0.28)	0.58 (0.50)	0.041
VCDR [IQR]	0.26 [0.16, 0.37]	0.26 [0.16, 0.38]	0.30 [0.20, 0.40]	0.33 [0.24, 0.41]	0.34 [0.18, 0.43]	0.27 [0.12, 0.42]	0.018
Longer disc diameter (mm)	1.74 (0.16)	1.74 (0.18)	1.76 (0.19)	1.72 (0.17)	1.72 (0.18)	1.81 (0.37)	0.604
Shorter disc diameter (mm)	1.55 (0.15)	1.56 (0.16)	1.56 (0.16)	1.56 (0.17)	1.54 (0.18)	1.60 (0.29)	0.931
Optic disc area (mm^2^)	2.14 (0.40)	2.14 (0.43)	2.18 (0.45)	2.13 (0.42)	2.10 (0.45)	2.34 (0.96)	0.759
Torsion angle (absolute), [IQR]	21.00 [13.50, 31.00]	21.00 [13.00, 30.00]	19.00 [12.00, 25.00]	20.00 [14.00, 29.50]	20.00 [12.00, 30.00]	16.00 [12.25, 28.00]	0.663
Tilted	7 (3.6%)	7 (3.7%)	6 (5.0%)	1 (2.0%)	1 (2.6%)	0 (0.0%)	0.929
Torted	118 (67.4%)	116 (67.1%)	71 (65.1%)	27 (69.2%)	20 (60.6%)	4 (66.7%)	0.975

MRW, minimum rim width; pRNFL, peripapillary retinal nerve fibre layer; VCDR, vertical cup to disc ratio.

We captured optic disc photographs and manual measurements were conducted by an experienced masked investigator under supervision by an experienced ophthalmologist (ImageJ software, version 1.53a) as described previously.^6^ The following optic disc parameters were measured: vertical disc length (mm), vertical cup length (mm), VCDR, longer disc diameter (mm), shorter disc diameter (mm), optic disc area (mm^2^), torsion angle (absolute, in degrees), tilted disc, and torted disc. All measured lengths were corrected for ocular magnification, according to Garway-Heath et al.^25^

### Covariates

The covariates were defined as factors that may affect the main outcome measures, that is age (years), sex (female), spherical equivalent (diopters), axial length (mm), optic disc size (mm^2^), GA (weeks), birth weight (kg), birthweight percentile, ROP, ROP treatment, maternal smoking, placental insufficiency, preeclampsia, breastfeeding, and perinatal adverse events such as an intraventricular hemorrhage (at least grade 3 or parenchymal hemorrhage), necrotizing enterocolitis, and moderate or severe bronchopulmonary dysplasia (defined according to the definition of the German query for quality control of the neonatal clinics^28^).

### Statistical Analysis

The main outcome measures were the global pRNFL-thickness, the global MRW, the BMO area, and VCDR. Descriptive statistics were stratified according to the groups. Absolute and relative frequencies were calculated for dichotomous parameters, with the mean and standard deviation calculated for approximately normally distributed variables (otherwise median and interquartile range). One-way ANOVA was used to compare continuous and approximately normally distributed variables and the chi-squared test for dichotomous variables. The Kruskal-Wallis-Test was used to compare continuous variables of non-normal distribution. Linear regression with generalized estimating equations was conducted for pRNFL-thickness, global MRW, and BMO area analysis in a complete case analysis set to account for inter-eye correlations within each participant.

Because of the skewness in the VCDR distribution, the associations were analysed using a linear quantile mixed model with the eyes set as a random factor to account for the inter-eye correlation and the median (τ = 0.5) was used to calculate the estimator. Univariable analysis was performed regarding the influence of GA [groups: GA ≤ 28 weeks, GA 29–32 weeks, GA 33–36 weeks, GA ≥ 37 weeks (Reference)] , birth weight percentile [groups: <25, 25–75 (Reference), >75], ROP [groups: treated ROP, ROP, no ROP (Reference)], perinatal adverse events, preeclampsia, placental insufficiency, maternal smoking during pregnancy, and breastfeeding. The multivariable model (model 1) additionally adjusted the analyses for age (years), sex (female), axial length (mm), and optic disc area (mm^2^) for the VCDR analysis. An additional multivariable sensitivity analysis was performed after exclusion of participants with periventricular leukomalacia (PVL) (excluded were *n* = 2 in the group with GA 29–32 weeks, *n* = 6 in the group with GA ≤ 28 weeks without ROP, *n* = 5 in preterms with ROP without treatment, and *n* = 2 in preterm children with treated ROP).

The influence of perinatal factors on the pRNFL sectors, the global pRNFL and MRW as well as the BMO was analyzed using a multivariable analysis model adjusted for age (years), sex (female), GA (groups), birthweight percentile (groups) and axial length (mm) and sequentially each of the following variable separately: ROP (groups), perinatal adverse events, maternal smoking during pregnancy, preeclampsia, placental insufficiency, and breastfeeding.

Because this is an explorative study, a significance level was not defined and no adjustment for multiple testing was conducted; thus *P* values were reported only for descriptive purposes and should be interpreted with caution.^29^ Calculations were performed using R (R Core Team [2021]; R: A language and environment for statistical computing. R Foundation for Statistical Computing, Vienna, Austria, URL https://www.R-project.org/, R version 4.1.2 [2021-11-01]).

## Results

### Participant Characteristics

This study included 1546 eyes of 793 participants born preterm and full-term (mean age = 11.41 years, 418 female subjects). Overall, 502 eyes of 257 participants with a GA ≥ 37 weeks (group 1, control group), 474 eyes of 240 participants with a GA between 33 and 36 weeks without ROP (group 2), 318 eyes of 164 participants with a GA between 29 and 32 weeks without ROP (group 3), 136 eyes of 70 participants with a GA ≤ 28 weeks without ROP (group 4), 99 eyes of 53 participants with a GA between 24 and 32 weeks with ROP without treatment (group 5), and 17 eyes of nine participants with a GA between 24 and 32 weeks and with postnatal laser or anti-VEGF treatment for ROP (group 6) were assessed and their characteristics are summarized in Table 1. Of the participants treated for ROP, one received anti-VEGF treatment whereas the others received photocoagulation treatment.

### Descriptive Sectorial Papillary Measures

Descriptive sectorial measurements for pRNFL, BMO area, and MRW are displayed in Table 2 with graphs presented in the Figure and Supplementary Figure S2. In multivariable linear regression analyses GA ≤ 28 weeks was associated with thinner pRNFL globally (β = −9.25, *P* < 0.001) and in all sectors with exception of the temporal one (all *P* < 0.001). GA between 29 and 32 weeks was only associated with the thinning of the temporal (β = −2.83, *P* = 0.02) and nasal sector (β = −3.49, *P* = 0.03). A birth weight percentile <25 was also associated with thinning of all sectors as well as globally, apart from the temporal sector (global β = −3.77, *P* < 0.001). Furthermore, ROP treatment was associated with a thicker pRNFL in the temporal (β = 43.31, *P* < 0.001) and inferotemporal sectors (β = 20.97, *P* = 0.04). Perinatal adverse events were associated with a thinner pRNFL globally (β = −7.68, *P* < 0.001) and in all three nasal sectors (*P* < 0.001). Maternal smoking during pregnancy was associated with thinner inferonasal pRNFL (β = −12.02, *P* = 0.003) and breastfeeding was associated with a thicker global pRNFL (β = 2.25, *P* = 0.02), as well as in the inferonasal and superonasal sectors. Results from the pRNFL-association analyses are displayed in Table 3.

**Figure.**
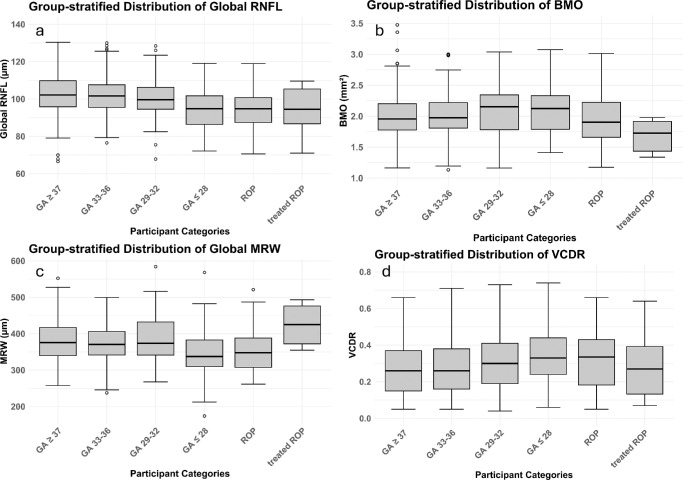
Descriptive group-stratified distribution of the optic nerve head parameters. **(a)** Global retinal nerve fiber layer. **(b)** Area of the Bruch's membrane opening. **(c)** Global minimum rim width. **(d)** Vertical cup-to-disc ratio in both eyes. BMO, Bruch's membrane opening; GA, gestational age; MRW, minimum rim width; RNFL, here: peripapillary retinal nerve fibre layer; ROP, retinopathy of prematurity; VCDR, vertical cup to disc ratio.

**Table 3.** Association Analyses of the Peripapillary Retinal Nerve Fiber Layer

	Global		Superotemporal		Temporal		Inferotemporal		Inferonasal		Nasal		Superonasal	
Factor	**β** (95% CI)	*P*	**β** (95% CI)	*P*	**β** (95% CI)	*P*	**β** (95% CI)	*P*	**β** (95% CI)	*P*	**β** (95% CI)	*P*	**β** (95% CI)	*P*
Age (y)	0.13 [−0.15; 0.41]	0.35	0.23 [−0.21; 0.77]	0.40	−0.38 [−0.69; −0.07]	0.02	0.11 [−0.40; 0.62]	0.67	0.11 [−0.56; 0.78]	0.75	0.25 [−0.14; 0.64]	0.21	0.64 [0.08; 1.20]	0.03
Sex (Female)	−0.16 [−1.92; 1.61]	0.86	0.20 [−3.29; 3.70]	0.91	1.31 [−0.71; 3.33]	0.20	1.36 [−1.85; 4.57]	0.41	−3.13 [−7.03; 0.76]	0.11	−0.02 [−2.43; 2.39]	0.99	−1.95 [−5.61; 1.71]	0.30
Axial Length	0.13 [−0.89; 1.15]	0.80	2.09 [0.28; 3.91]	0.02	1.63 [0.35; 2.92]	0.01	0.28 [−1.67; 2.22]	0.78	−3.88 [6.07; −1.69]	<0.001	0.34 [−1.00; 1.68]	0.62	−0.16 [−2.24; 1.93]	0.88
Gestational age														
≤28wk	−9.25 [−12.07; −6.44]	<0.001	−11.12 [−16.23; −6.00]	<0.001	−2.65 [−6.23; 0.94]	0.15	−11.12 [−16.63; −5.61]	<0.001	−16.88 [−23.26; −10.50]	<0.001	−8.85 [−12.67; −5.04]	<0.001	−11.48 [−16.99; −5.97]	<0.001
29–32 wk	−1.64 [−3.90; 0.61]	0.15	2.22 [−2.13; 6.57]	0.32	−2.83 [−5.29; −0.38]	0.02	1.97 [2.13; 6.06]	0.35	−4.60 [−10.02; 0.82]	0.10	−3.49 [−6.62; −0.35]	0.03	0.51 [−4.02; 5.05]	0.82
33–36 wk	−0.37 [−2.38; 1.65]	0.72	1.09 [−2.92; 5.09]	0.59	−2.18 [−4.45; 0.08]	0.06	−1.39 [−4.88; 2.10]	0.44	−0.86 [−5.77; 4.05]	0.73	0.25 [−2.64; 3.14]	0.87	2.22 [−2.17; 6.60]	0.32
≥37 wk	Ref.	Ref.	Ref.	Ref.	Ref.	Ref.	Ref.	Ref.	Ref.	Ref.	Ref.	Ref.	Ref.	Ref.
Birth weight percentile														
<25	−3.77 [−5.59; −1.95]	<0.001	−4.77 [−8.38; −1.16]	0.01	−0.57 [−2.73; 1.59]	0.60	−3.44 [−6.84; −0.04]	0.05	−6.29 [−10.45; −2.14]	0.003	−3.43 [−5.91; −0.94]	0.007	−7.59 [−11.38; −3.79]	<0.001
25–75	Ref.	Ref.			Ref.	Ref.								
>75	0.33 [−2.41; 3.07]	0.81	−3.25 [−8.42; 1.92]	0.22	−1.03 [−4.27; 2.22]	0.54	1.51 [−3.11; 6.12]	0.52	4.43 [−2.94; 11.80]	0.24	2.84 [−1.17; 6.85]	0.17	−3.33 [−8.42; 1.75]	0.20
ROP														
ROP treatment (yes)	5.90 [−3.45; 15.24]	0.22	−6.83 [−21.16; 7.51]	0.35	43.31 [20.12; 66.50]	<0.001	20.97 [1.14; 40.81]	0.04	−22.90 [−47.63; 1.83]	0.07	−3.71 [−14.30; 6.87]	0.49	−26.22 [−34.37; −18.06]	<0.001
ROP (yes)	−0.27 [−3.73; 3.19]	0.88	−2.45 [−9.78; 4.88]	0.51	−0.23 [−3.81; 3.34]	0.90	−2.83 [−9.81; 4.14]	0.43	−4.44 [−12.55; 3.66]	0.28	1.91 [−3.73; 7.55]	0.51	2.71 [−5.52; 10.95]	0.52
No ROP	Ref.	Ref.	Ref.	Ref.	Ref.	Ref.	Ref.	Ref.	Ref.	Ref.	Ref.	Ref.	Ref.	Ref.
Perinatal adverse events (yes)	−7.68 [−11.74; −3.63]	<0.001	−5.97 [−13.41; 1.47]	0.12	1.10 [−4.99; 7.19]	0.72	−4.55 [−12.59; 3.48]	0.27	−22.01 [−29.91; −14.11]	<0.001	−8.61 [−13.21; −4.02]	<0.001	−13.66 [−21.96; −5.35]	0.001
Preeclampsia (Yes)	−0.78 [−3.92; 2.35]	0.62	−4.20 [−9.55; 1.16]	0.12	−0.75 [−4.55; 3.05]	0.70	−3.79 [−9.94; 2.36]	0.23	1.61 [−5.66; 8.89]	0.66	2.77 [−1.77; 7.31]	0.23	−4.76 [−9.86; 0.34]	0.07
Placental insufficiency (Yes)	−3.95 [−9.15; 1.26]	0.14	−5.72 [−14.03; 2.59]	0.18	−3.67 [−9.60; 2.26]	0.22	−5.47 [−15.67; 4.73]	0.29	−5.43 [−16.69; 5.83]	0.34	0.01 [−5.98; 5.99]	1.00	−7.47 [−15.32; 0.37]	0.06
Smoking during pregnancy (Yes)	−4.00 [−8.11; 0.11]	0.06	1.59 [−6.34; 9.51]	0.69	−2.72 [−7.90; 2.46]	0.30	−4.48 [−12.73; 3.77]	0.29	−12.02 [−19.97; −4.08]	0.003	−4.54 [−8.82; −0.26]	0.04	−2.33 [−9.50; 4.84]	0.52
Breastfeeding (Yes)	2.25 [0.44; 4.06]	0.02	3.56 [−0.10; 7.21]	0.06	0.36 [−1.90; 2.61]	0.76	1.45 [−1.96; 4.85]	0.41	6.27 [2.25; 10.29]	<0.002	0.65 [−1.88; 3.18]	0.61	4.84 [1.09; 8.60]	0.01

Linear regression analysis using generalized estimating equations to control for correlations between OD and OS. Model 1 included age (years), sex (female), axial length (mm), gestational age (categories), and birthweight percentile (categories). In a stepwise analysis, the following factors were included separately in model 1 (ROP [categorical], perinatal adverse events, preeclampsia, placental insufficiency, smoking during pregnancy, and breastfeeding) and are reported below model 1.

Association analyses with global MRW in the multivariable model, adjusted for age, sex and axial length did not show any association with different perinatal parameters. However, in temporal sectors, individuals treated for ROP showed a larger MRW (inferotemporal β = 75.99, temporal β = 97.44 and superotemporal β = 74.40, all *P* < 0.008). Furthermore, individuals born extremely preterm showed a smaller MRW in both inferior sectors (inferotemporal β = −30.36 and inferonasal β = −28.85, all *P* ≤ 0.02). Association analyses of BMO-area revealed only a marginal association with GA 29–32 weeks when adjusted for age, sex, and axial length (β = 0.13; 95% CI, 0.01–0.25; *P* = 0.04) (Table 4).

**Table 4.** Association Analyses of the Minimum Rim Width and Bruch's Membrane Opening

Factor	Global		Superotemporal		Temporal		Inferotemporal		Inferonasal		Nasal		Superonasal		BMO (mm^2^)	
	**β** (95% CI)	*P*	**β** (95% CI)	*P*	**β** (95% CI)	*P*	**β** (95% CI)	*P*	**β** (95% CI)	*P*	**β** (95% CI)	*P*	**β** (95% CI)	*P*	**β** (95% CI)	*P*
Age, y	−1.47 [−3.58; 0.64]	0.17	−2.08 [−4.47; 0.31]	0.09	−2.24 [−4.11; −0.36]	0.02	−0.85 [−3.13; 1.44]	0.47	−1.92 [−4.39; 0.56]	0.13	−1.27 [−3.95; 1.40]	0.35	−0.54 [−3.56; 2.49]	0.73	−0.003 [−0.02; 0.013]	0.69
Sex (Female)	−5.41 [−18.02 7.20]	0.4	4.04 [−9.76; 17.83]	0.57	−9.30 [−20.43; 1.83]	0.10	−1.10 [−14.75; 12.56]	0.87	−4.57 [−19.74; 10.60]	0.56	−5.90 [−21.58; 9.78]	0.46	−7.19 [−24.08; 9.70]	0.40	−0.05 [−0.14; 0.04]	0.24
Axial Length	−2.49 [−4.69; −0.28]	0.03	−1.44 [−2.34; −0.53]	0.002	0.80 [0.25; 1.39]	0.007	−5.45 [−9.28; −1.62]	0.005	−5.23 [−9.14; −1.33]	0.009	−0.83 [−3.62; 1.95]	0.56	−4.60 [−9.94; 0.75]	0.09	−0.01 [−0.05; 0.03]	0.66
Gestational age																
≤28 weeks	−15.88 [−36.15; 4.38]	0.125	−16.14 [−35.76; 3.47]	0.11	0.66 [−17.81; 19.13]	0.94	−30.36 [−51.32; −9.39]	0.005	−28.85 [−53.93; −3.78]	0.02	−16.13 [−41.50; 9.23]	0.21	−20.85 [−45.99; 4.29]	0.10	−0.01 [−0.13; 0.12]	0.93
29–32 weeks	−0.95 [−18.79; 16.88]	0.92	2.35 [−17.57; 22.27]	0.82	1.27 [−12.82; 15.36]	0.86	−0.86 [−19.99; 18.27]	0.93	−0.40 [−21.60; 20.80]	0.97	−4.47 [−27.46; 18.52]	0.70	1.74 [−22.57; 26.05]	0.89	0.13 [0.01; 0.25]	0.04
33–36 weeks	−6.17 [−19.73; 7.38]	0.37	−12.04 [−29.02; 4.94]	0.16	−5.60 [−18.43; 7.23]	0.39	−8.73 [−24.46; 7.01]	0.28	−0.77 [−17.41; 15.87]	0.93	−3.90 [−19.99; 12.19]	0.63	−10.36 [29.76; 9.04]	0. 30	0.02 [−0.08; 0.11]	0.73
≥37 weeks	Ref.	Ref.	Ref.	Ref.	Ref.	Ref.	Ref.	Ref.	Ref.	Ref.	Ref.	Ref.	Ref.	Ref.	Ref.	Ref.
Birth weight percentile																
<25	−5.51 [−18.82; 7.81]	0.42	−2.20 [−17.41; 13.00]	0.78	0.81 [−10.82; 12.43]	0.89	−1.55 [−15.91; 12.82]	0.83	−11.55 [−27.31; 4.22]	0.15	−10.23 [−26.77; 6.32]	0.23	−6.73 [−24.74; 11.27]	0.46	0.02 [−0.07; 0.11]	0.68
25–75	Ref.	Ref.			Ref.	Ref.									Ref.	Ref.
>75	−5.17 [−27.79; 17.44]	0.65	−12.85 [−35.89; 10.19]	0.27	−9.48 [−29.54; 10.59]	0.35	7.00 [−20.14; 34.14]	0.61	11.00 [−17.85; 39.86]	0.45	−9.32 [−37.94; 19.30]	0.52	−5.59 [−33.09; 21.91]	0.69	−0.004 [−0.16; 0.15]	0.95
ROP																
ROP treatment	61.92 [−0.42; 124.25]	0.05	74.40 [25.30; 123.50]	0.003	97.44 [34.11; 160.77]	0.003	75.99 [20.78; 131.2]	0.007	50.44 [−0.93; 101.81]	0.05	34.04 [−53.39; 121.47]	0.45	48.62 [−23.43; 120.66]	0.19	−0.34 [−0.72; 0.03]	0.07
ROP	0.57 [−9.30; 10.43]	0.91	0.04 [−3.50; 3.58]	0.98	−0.32 [−1.61; 0.97]	0.63	3.09 [−12.24; 18.42]	0.69	1.49 [−17.16; 20.15]	0.88	−1.19 [−14.23; 11.86]	0.86	−1.61 [−22.46; 19.23]	0.88	0.12 [−0.30; 0.55]	0.57
No ROP	Ref.	Ref.	Ref.	Ref.	Ref.	Ref.	Ref.	Ref.	Ref.	Ref.	Ref.	Ref.	Ref.	Ref.	Ref.	Ref.
Perinatal adverse events	−24.18 [−52.91; 4.55]	0.10	−24.34 [−52.55; 3.88]	0.09	−18.63 [−44.82; 7.56]	0.16	−9.33 [−36.74; 18.09]	0.51	−21.08 [−55.85; 13.68]	0.23	−32.37 [−67.85; 3.11]	0.07	−28.65 [−64.34; 7.05]	0.12	−0.15 [−0.32; 0.02]	0.08
Preeclampsia	−4.76 [−26.71; 17.20]	0.67	−10.51 [−33.19; 12.17]	0.36	4.24 [−16.10; 24.57]	0.68	−3.51 [−25.51; 18.49]	0.75	−9.62 [−35.64; 16.40]	0.47	−4.79 [−33.50; 23.91]	0.74	−16.04 [−42.37; 10.29]	0.23	−0.09 [−0.22; 0.05]	0.22
Placental insufficiency	−1.47 [−35.64; 32.71]	0.93	0.49 [−34.68; 35.67]	0.98	−1.91 [−36.62; 32.80]	0.91	−0.553 [−30.45; 29.34]	0.97	3.77 [−36.92; 44.45]	0.86	−6.59 [−47.90; 34.72]	0.75	8.09 [−29.33; 45.52]	0.67	−0.18 [−0.40; 0.04]	0.1
Smoking during pregnancy	2.97 [−22.64; 28.59]	0.82	13.41 [−14.72; 41.55]	0.35	21.82 [−4.75; 48.39]	0.11	17.19 [−15.16; 49.55]	0.30	−7.29 [−39.09; 24.50]	0.65	−17.38 [−46.04; 11.29]	0.23	3.94 [−30.12; 37.99]	0.82	0.07 [−0.16; 0.29]	0.56
Breastfeeding	−5.85 [−19.84; 8.15]	0.41	−16.04 [−31.45; −0.63]	0.04	−8.73 [−21.46; 4.00]	0.18	−0.25 [−15.66; 15.16]	0.97	1.72 [−15.45; 18.89]	0.84	−4.08 [−21.30; 13.15]	0.64	−7.29 [−26.20; 11.63]	0.45	0.07 [−0.02; 0.16]	0.12

Linear regression analysis using generalized estimating equations to control for correlations between OD and OS. Model 1 included age (years), sex (female), axial length (mm), gestational age (categories), and birth weight percentile (categories). In a stepwise analysis, the following factors were included separately in model 1 (ROP [categorical], perinatal adverse events, preeclampsia, placental insufficiency, smoking during pregnancy, and breastfeeding) and are reported below model 1.

### Linear Quantile Mixed Model Association Analyses of the Vertical Cup-to-Disc Ratio

The linear quantile mixed model showed that VCDR was associated with extremely preterm birth (β = 0.05; 95% CI, 0.003–0.09; *P* = 0.04), as well as moderate preterm birth (β = 0.05; 95% CI, 0.009–0.09; *P* = 0.02) and preeclampsia (β = 0.05; 95% CI, 0.019–0.08; *P* < 0.001) in the univariable analyses (Supplementary Table S1). The association with extreme prematurity (β = 0.05; 95% CI, 0.02–0.08; *P* = 0.001) and moderate prematurity (β = 0.04; 95% CI, 0.01–0.06; *P* = 0.004) persisted, and perinatal adverse events (β = 0.05; 95% CI, 0.01–0.09; *P* = 0.02) were also associated with a larger VCDR after adjustment in the multivariable analyses adjusted for sex, age, axial length and optic disc area. None of the other parameters revealed an association in multivariable analyses. A sensitivity analysis with exclusion of the 15 cases with PVL did show similar results.

## Discussion

This is the first study to comprehensively assess the optic nerve head characteristics pRNFL, BMO area, MRW, and VCDR in a combined approach in a large cohort of children aged four to 17 years of age with and without previous ROP and ROP treatment. This analysis revealed that the main perinatal parameters leading to a thinner pRNFL were extreme prematurity and a birth weight percentile <25, whereas individuals treated for ROP displayed a thicker pRNFL in temporal and inferotemporal sectors. Furthermore, individuals affected by adverse perinatal events had a thinner pRNFL in all nasal sectors, individuals who had records of maternal smoking had a thinner inferonasal pRNFL and individuals who were breastfed had greater pRNFL-thickness globally, as well as in the inferonasal and superonasal sectors.

The pRNFL results are in line with results of previous assessments in children^3^^,^^13^^,^^14^ and adults born preterm,^4^ which found the pRNFL to be thinner in all but temporal sectors. However, the current study results in children revealed that only extreme prematurity (GA ≤ 28 weeks) was associated with a thinner pRNFL. This may be explained by changes in neonatal care with the introduction of antenatal corticosteroids, surfactant therapy, and high-frequency ventilation,^30^ which could have prevented the impaired development of neuronal tissue measured by pRNFL thickness in individuals born only moderately preterm but not have had sufficient effect on children born extremely preterm. The effect estimators of GA on global pRNFL thickness support this hypothesis.^4^ The larger VCDR, which we observed in children born more preterm, is also in line with the general thinning of the pRNFL in those individuals and has also been described in previous studies of children and adults.^6^^,^^13^ This analysis suggested that median VCDR was only associated with extreme and moderate prematurity, as well as perinatal adverse events after adjustment.^31^

The results regarding a thinner pRNFL with extreme prematurity are also in line with the MRW results, which tended to be thinner in children born extremely preterm. However, this was only significant in inferior sectors (inferotemporal, inferonasal) but is in line with the results by Wenner et al.^13^ who reported a significantly thinner rim area in children born preterm and an even smaller rim area in children born preterm with brain injury, after adjusting for spherical equivalent, birth weight, GA, bronchopulmonary dysplasia and ROP. The present study also revealed that individuals who experienced perinatal adverse events (comprising individuals with intraventricular hemorrhage) had a thinner pRNFL globally, especially in the nasal sectors. Both perinatal adverse events as well as maternal smoking affect neurodevelopment^32^^,^^33^ which might indirectly affect the pRNFL thickness, whereas breastfeeding had positive effects on brain development in a previous study,^34^ possibly explaining the findings with respect to pRNFL thickness. One mechanism behind the changes in optic nerve head anatomy in extremely preterm children could be retrograde transynaptical degeneration, which, following neurological injuries due to preterm birth such as PVL or other smaller white matter injuries, might explain the reason for the thinner pRNFL.^35^ VCDR analyses did not differ after exclusion of participants with PVL, highlighting the sole effects of immaturity. This is supported by further research showing that even children born preterm without major cerebral pathology revealed a decrease in both gray and white matter tissue.^31^

Regarding the BMO area, Lee et al.^14^ found no correlation between GA and BMO diameter in their group of preterm individuals. Likewise, the BMO area in the present study was only marginally associated with moderately preterm birth but no other factors tested, suggesting that BMO is independent of prenatal factors including GA and birth weight.

A thicker pRNFL was observed in the temporal sectors of individuals treated for ROP in both adults and now in children, which is in line with the results from previous studies,^14^^,^^36^^,^^37^ although not all studies differentiated between ROP and ROP treatment. Furthermore, individuals treated for ROP showed a significantly larger MRW in all temporal sectors in our analyses, which supports the findings of a larger pRNFL. This alteration might be the result of a redistribution from the superior sector (which was also reduced in our cohort) to the temporal sector along with the migration disorder of the macula and increased foveal thickness.^4^^,^^38^^,^^39^

### Strengths and Limitations

This study is limited by its single-center and hospital-based design. Not all participants who were approached participated in the study, and missing contact details might have resulted in selection bias. Furthermore, some of the younger children especially children with extreme prematurity might have had problems with fixation, which could have biased our assessments. Although children with treated ROP were examined, this group is rather small, and there were different types of treatments (laser-treatment vs. anti-VEGF medication) within the group, although most children received treatment by photocoagulation. It also needs to be acknowledged that groups with treated ROP had higher stages of ROP and higher proportions of Plus Disease, which may have influenced the results more than the actual treatment. A sensitivity analysis with exclusion of participants with plus disease was not conducted because of the low number of affected participants which could also limit our analysis. Furthermore, the number of participants who were affected by bronchopulmonary dysplasia, intraventricular hemorrhage, and necrotizing enterocolitis was low, and therefore we summarized these as adverse events instead of assessing the effects for each event solely. Unfortunately, we are therefore unable to draw any conclusions about the effects of the individual diseases. Furthermore, the proportion of images that could not be assessed was rather high in the groups with ROP and treated ROP, which is a result of fixation instabilities and a higher proportion of developmental delays in these groups. Additionally, children born preterm have increased refractive errors which could also limit our analyses, although we adjusted our analyses for axial length and there were no cases of extremely high myopia in this cohort. The range of spherical equivalent in our included participants was −10.79 D to 7.40 D; therefore our findings may not necessarily generalize to children with even higher degrees of ametropia. Also, because prematurity, low birthweight, and several other parameters are interrelated, we performed multivariable analyses adjusting for these potential confounders. Although the association between RNFL thickness and breastfeeding remained significant, residual bias cannot be fully excluded, and the results should be interpreted with caution.

Nonetheless, this is one of the largest cohorts assessing children over a wide age span with and without ROP and ROP treatment, enabling the investigation of children born preterm without ROP. Because this is a combined prospective and retrospective cohort study, perinatal factors were assessed and integrated into the analyses of current outcomes. Investigators were blinded, and data quality was ensured by standardized examination and analysis procedures to minimize bias.

## Conclusions

In conclusion, the results of this study suggest that prematurity, especially extreme prematurity, leads to a thinner pRNFL and a larger VCDR measure, and in turn, advanced ROP stages and need for ROP treatment lead to a thicker pRNFL in temporal sectors in line with MRW results, indicating that children after treatment for ROP may have a thicker MRW in all temporal sectors. In addition, the results showed that in this study, perinatal adverse events and maternal smoking during pregnancy negatively affected pRNFL thickness, suggesting a reduced pRNFL capacity and a potentially greater risk of developing age-related neurodegenerative diseases in these cases, whereas children who were breastfed as infants had a thicker pRNFL and therefore may have a greater reserve pRNFL capacity to protect against age-related pRNFL thinning.

## Supplementary Material

Supplement 1
