# Supplementary material for: Optic Disc Characteristics in Children Born Preterm With and Without ROP: Results From the Gutenberg Prematurity Eye Study Young (GPESY)
Source: Invest Ophthalmol Vis Sci. 2025 Oct 9;66(13):21. doi: 10.1167/iovs.66.13.21 (PMC12517363; doi:10.1167/iovs.66.13.21)
Supplement: Supplement 1 [file iovs-66-13-21_s001.docx]

Supplemental Figure S1. Design of the Gutenberg Prematurity Eye Study Young (GPESY)


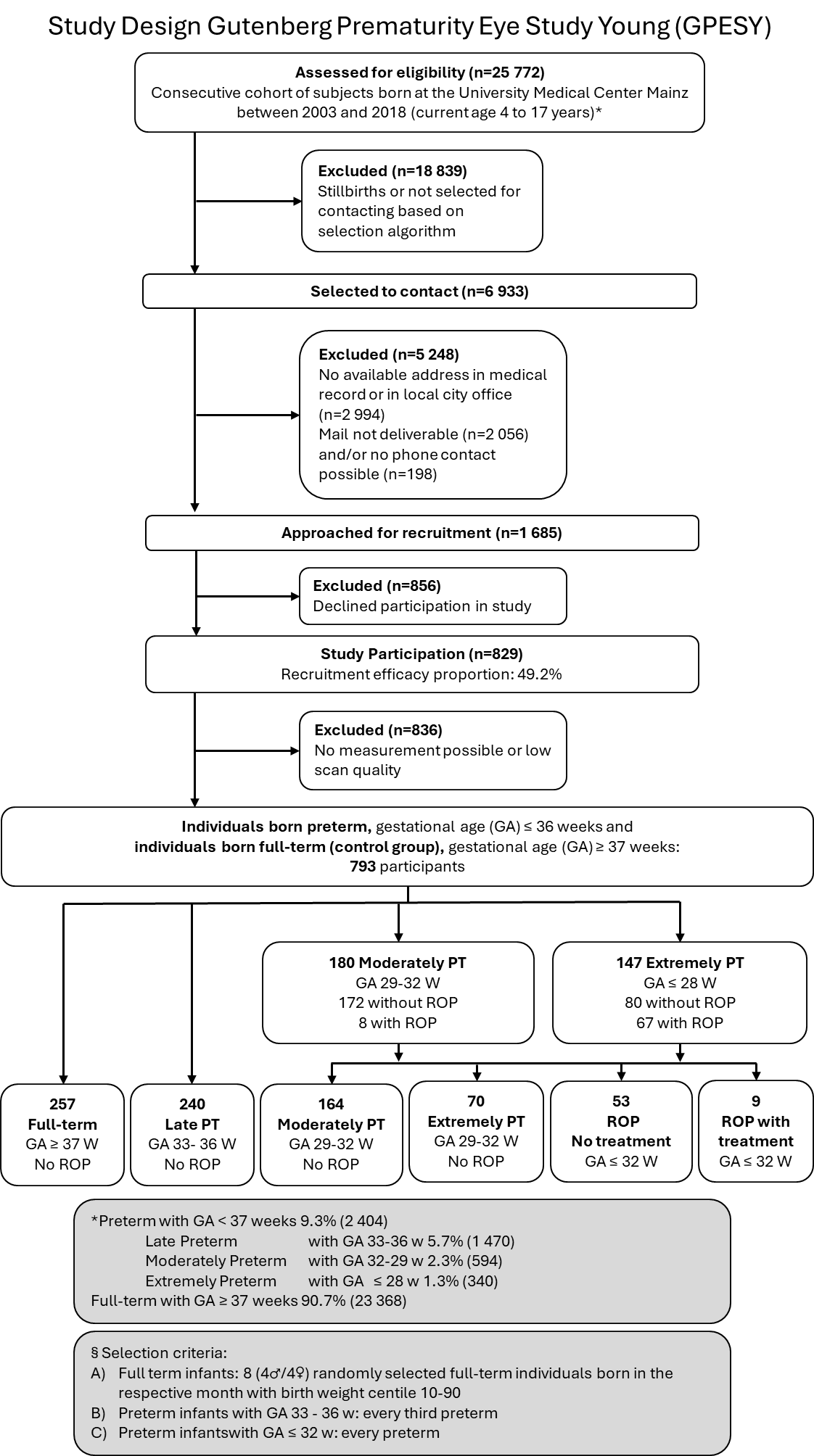


GA – Gestational age, ROP – Retinopathy of prematurity, PT - preterm

Supplemental Figure S2. Descriptive group-stratified distribution of the optic nerve head parameters related to birth weight percentile


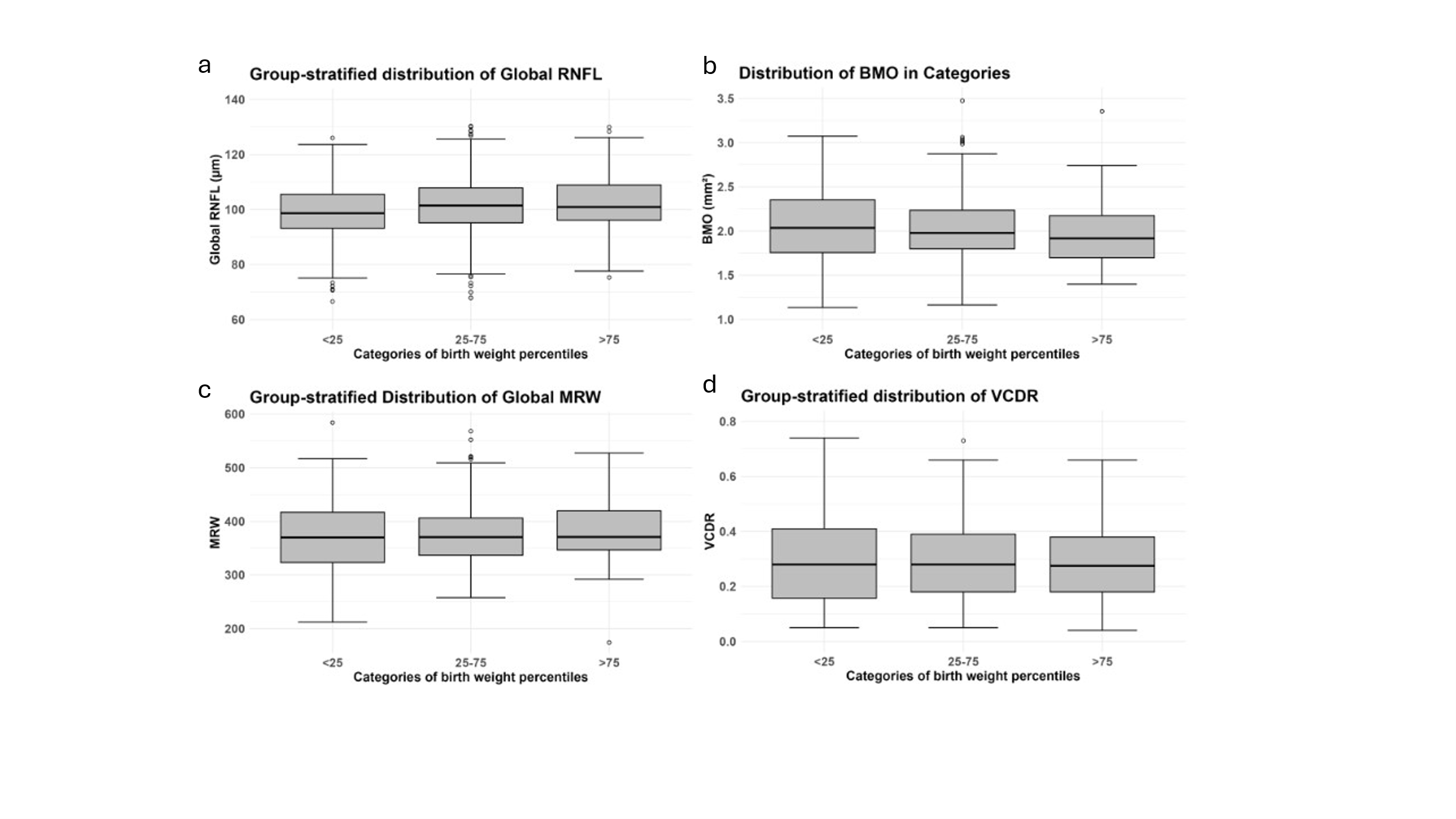


(a) global retinal nerve fibre layer, (b) Area of the Bruch’s Membrane Opening, (c) global minimum rim width and (d) vertical cup-to-disc ratio in both eyes.

#### RNFL – retinal nerve fiber layer, BMO – Bruch’s membrane opening, MRW – minimum rim width, VCDR – vertical cup-to-disc ratio

Supplemental Figure S3. Minimum Rim Width and Bruch’s Membrane Opening – Image output


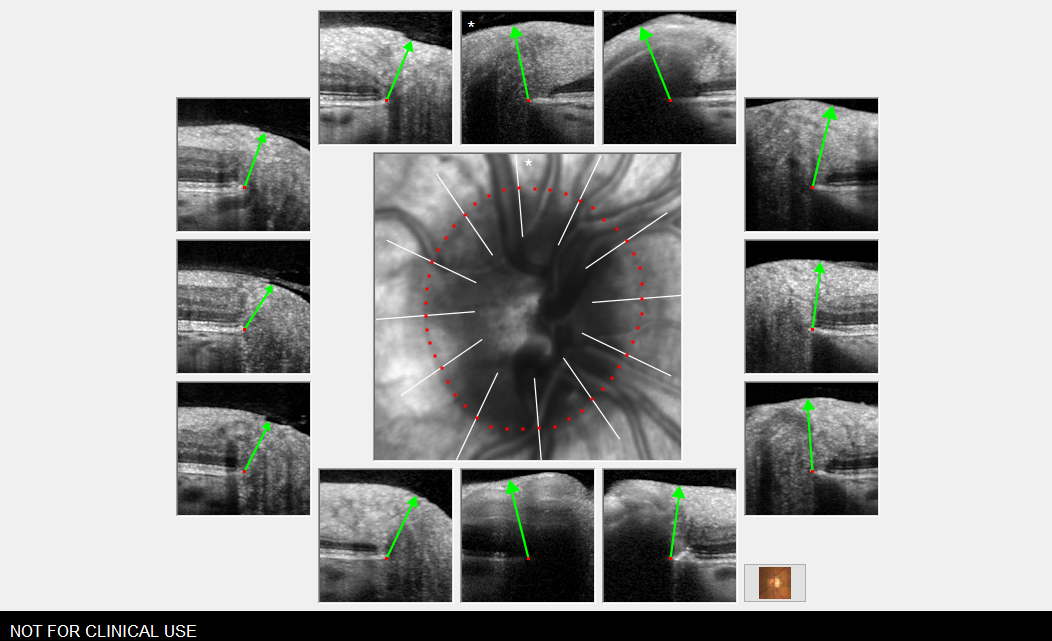


Red dotted line – Bruch’s Membrane Opening, The green arrows depict the measurements of the Minimum Rim Width

|  | **Univariable** |  | **Model 1** |  |
| --- | --- | --- | --- | --- |
| **VCDR** | β (median) [95%-CI] | p | β (median) [95%-CI] | p |
| n=571 |  |  |  |  |
| Gestational age |  |  |  |  |
| ≤28 wk | 0.05 [0.003;0.09] | **0.04** | 0.05 [0.02;0.08] | **0.001** |
| 29–32 wk | 0.05 [0.009;0.09] | **0.02** | 0.04 [0.01;0.06] | **0.004** |
| 33–36 wk | -0.001 [-0.04;0.04] | 0.95 | 0.001 [-0.02;0.02] | 0.92 |
| ≥37 wk | Ref. |  |  |  |
| Birth weight percentile |  |  |  |  |
| <25 | 0.001 [-0.04;0.04] | 0.97 | 0.01 [-0.02;0.04] | 0.51 |
| 25–75 | Ref. |  |  |  |
| >75 | -0.005 [-0.05;0.04] | 0.83 | -0.01 [-0.05;0.02] | 0.39 |
| ROP |  |  |  |  |
| ROP treatment (yes) | -0.04 [-0.14;0.05] | 0.37 | -0.02 [-0.07;0.03] | 0.39 |
| ROP (yes) | 0.05 [-0.01;0.10] | 0.10 | 0.04 [-0.005;0.09] | 0.08 |
| No ROP | Ref. |  |  |  |
| Perinatal adverse events (yes) | 0.03 [-0.02; 0.09] | 0.23 | 0.05 [0.01; 0.09] | **0.02** |
| Preeclampsia (yes) | 0.05 [0.019; 0.08] | **<0.001** | 0.02 [-0.008; 0.06] | 0.14 |
| Placental insufficiency (yes) | -0.004 [-0.1; 0.09] | 0.93 | 0.02 [-0.04; 0.09] | 0.48 |
| Smoking during pregnancy (yes) | -0.0003 [-0.06; 0.06] | 0.99 | -0.002 [-0.06; 0.05] | 0.95 |
| Breastfeeding (yes) | -0.002 [-0.03; 0.03] | 0.91 | -0.001 [0.03;0.03] | 0.93 |

Supplemental Table 1: Linear quantile mixed model association analyses of vertical cup-to-disc-ratio

VCDR – vertical cup-to-disc ratio, CI – confidence interval, ROP – retinopathy of prematurity, wk - weeks

Model 1: multivariable model, adjusted for age, sex, axial length, and optic disc area
